# Oosporein from *Tremella fuciformis*

**DOI:** 10.1107/S1600536812012950

**Published:** 2012-03-31

**Authors:** Gang He, Jun Yan, Xiao-Yong Wu, Xiao-Jun Gou, Wan-Chen Li

**Affiliations:** a Maize Research Institute, Sichuan Agricultural University, Chengdu 611130, People’s Republic of China; b The Key Laboratory of Medicinal and Edible Plants Resources Development of Sichuan Education Commission, Chengdu University, Chengdu 610106, People’s Republic of China

## Abstract

The title compound [systematic name: 3,3′,6,6′-tetra­hydroxy-4,4′-dimethyl-1,1′-bi(cyclo­hexa-3,6-diene)-2,2′,5,5′-tetra­one], C_14_H_10_O_8_, was isolated from *Tremella fuciformis*. The mol­ecule has 2 symmetry, with the mid-point of the C—C bond linking the cyclo­hexa­dienedione rings located on a twofold rotation axis. In the mol­ecule, the ring is approximately planar, with an r.m.s. deviation of 0.0093 Å, and the two rings make a dihedral angle of 67.89 (5)°. Inter­molecular O—H⋯O hydrogen bonding occurs in the crystal structure.

## Related literature

For general background to the title compound, see: Takeshita & Anchel (1965 ▶). For the chemical structure of the title compound established from NMR data, see: Richard *et al.* (1974 ▶).
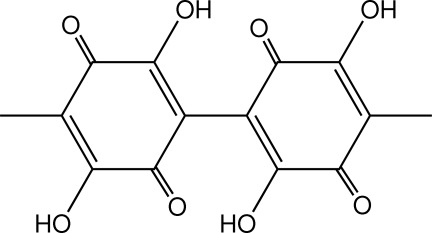


## Experimental

### 

#### Crystal data

C_14_H_10_O_8_
*M* *_r_* = 306.22
Monoclinic, 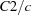

*a* = 11.9983 (9) Å
*b* = 8.2981 (6) Å
*c* = 13.7634 (11) Å
β = 105.994 (7)°
*V* = 1317.28 (17) Å^3^
*Z* = 4
Mo *K*α radiation
μ = 0.13 mm^−1^
*T* = 293 K
0.25 × 0.20 × 0.20 mm

#### Data collection

Oxford Diffraction Xcalibur Eos diffractometer
2848 measured reflections
1342 independent reflections
937 reflections with *I* > 2σ(*I*)
*R*_int_ = 0.022

#### Refinement

*R*[*F* ^2^ > 2σ(*F* ^2^)] = 0.044
*wR*(*F* ^2^) = 0.118
*S* = 1.06
1342 reflections
103 parameters
H-atom parameters constrained
Δρ_max_ = 0.21 e Å^−3^
Δρ_min_ = −0.17 e Å^−3^

### 

Data collection: *CrysAlis CCD* (Oxford Diffraction, 2009 ▶); cell refinement: *CrysAlis CCD*; data reduction: *CrysAlis RED* (Oxford Diffraction, 2009 ▶); program(s) used to solve structure: *SHELXTL* (Sheldrick, 2008 ▶); program(s) used to refine structure: *SHELXTL*; molecular graphics: *SHELXTL*; software used to prepare material for publication: *SHELXTL*.

## Supplementary Material

Crystal structure: contains datablock(s) I, global. DOI: 10.1107/S1600536812012950/xu5486sup1.cif

Structure factors: contains datablock(s) I. DOI: 10.1107/S1600536812012950/xu5486Isup2.hkl

Supplementary material file. DOI: 10.1107/S1600536812012950/xu5486Isup3.cml

Additional supplementary materials:  crystallographic information; 3D view; checkCIF report

## Figures and Tables

**Table 1:** Hydrogen-bond geometry (Å, °)

*D*—H⋯*A*	*D*—H	H⋯*A*	*D*⋯*A*	*D*—H⋯*A*
O2—H2⋯O4^i^	0.82	2.03	2.770 (2)	150
O3—H3⋯O1^ii^	0.82	2.03	2.7658 (19)	150

Symmetry codes: (i) 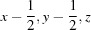; (ii) 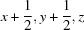.

**Fig. 1.**
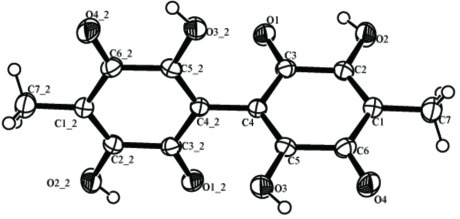
The molecular structure of the title compound with 30% probability displacement ellipsoids for non-H atoms.

## supplementary crystallographic information

### Comment

The oosporein was previously isolated from *Phlebia mellea* (Takeshita & Anchel, 1965), and its structure was established from the spectroscopic data (Richard *et al.*, 1974). In our recent investigation, it was isolated from *Tremella fuciformis* for the first time, and its structure is reported here.

The molecular structure of the title compound is shown in Fig. 1. The molecule of the title compound contains two plane six-membered rings which assumes a screw-plane conformation, and there is a dihedral angle between the two planes.

The crystal structure contains intermolecular O—H···O hydrogen bonding between the hydroxy group and the aldehyde atom (Table 1).

### Experimental

*Tremella fuciformis* was a culture collection of our laboratory, the stock culture was maintained on potato dextrose agar (PDA) slants and subcultured once a month. It was used in submerged culture. Agar, slants containing potato–dextrose–agar were inoculated with mycelia and incubated at 25 °C for 5 days, and then used as inoculums for seed culture. The seed culture was grown in 250 ml shake flasks containing 50 ml for 2 days at initial pH 6.8–7.0, 25 °C, and 150 rpm with a medium containing 20 g.l^-1^ glucose, 2 g.l^-1^ soybean meal leaching solution, 1.0 g.l^-1^ MgSO_4_, 1.0 g.l^-1^ KH_2_PO_4_ and 0.46 g.l^-1^ K_2_HPO_4_. Submerged fermentation was the same as the seed culture medium. All media were sterilized at 115 °C for 20 min. Fermentation liquid centrifugal (10000 rpm) and then rotary evaporation at 50 °C. Fermented liquid was concentrated by rotating evaporation at 50°C and add 4 times the volume of the anhydrous alcohol, 4 °C for the night precipitation, 10000 rmp centrifugal remove polysaccharides and protein. Rotated evaporation and concentration, concentrate on D101 macroporous resin adsorption, first washed with distilled water, then elution with 95% ethanol elution, eluent was saved at 4°C, to obtain crud crystals, crud crystals washing was 95% ethanol, and then recrystallized in 95% ethanol solution.

### Refinement

H atoms were located geometrically with O—H = 0.82 Å and C—H = 0.96 Å, and using a riding model with *U*_iso_(H) = 1.5*U*_eq_(C,*O*).

### Crystal data

**Table:**

C_14_H_10_O_8_	*F*(000) = 632
*M**_r_* = 306.22	*D*_x_ = 1.544 Mg m^−^^3^
Monoclinic, *C*2/*c*	Mo *K*α radiation, λ = 0.71073 Å
*a* = 11.9983 (9) Å	Cell parameters from 884 reflections
*b* = 8.2981 (6) Å	θ = 3.0–28.7°
*c* = 13.7634 (11) Å	µ = 0.13 mm^−^^1^
β = 105.994 (7)°	*T* = 293 K
*V* = 1317.28 (17) Å^3^	Block, colorless
*Z* = 4	0.25 × 0.20 × 0.20 mm

### Data collection

**Table:**

Oxford Diffraction Xcalibur Eos diffractometer	937 reflections with *I* > 2σ(*I*)
Radiation source: fine-focus sealed tube	*R*_int_ = 0.022
Graphite monochromator	θ_max_ = 26.4°, θ_min_ = 3.0°
Detector resolution: 10.0 pixels mm^-1^	*h* = −14→11
ω scans	*k* = −9→10
2848 measured reflections	*l* = −17→13
1342 independent reflections	

### Refinement

**Table:**

Refinement on *F*^2^	Primary atom site location: structure-invariant direct methods
Least-squares matrix: full	Secondary atom site location: difference Fourier map
*R*[*F*^2^ > 2σ(*F*^2^)] = 0.044	Hydrogen site location: inferred from neighbouring sites
*wR*(*F*^2^) = 0.118	H-atom parameters constrained
*S* = 1.06	*w* = 1/[σ^2^(*F*_o_^2^) + (0.0491*P*)^2^ + 0.4579*P*] where *P* = (*F*_o_^2^ + 2*F*_c_^2^)/3
1342 reflections	(Δ/σ)_max_ < 0.001
103 parameters	Δρ_max_ = 0.21 e Å^−^^3^
0 restraints	Δρ_min_ = −0.17 e Å^−^^3^

### Special details

**Table:**

Geometry. All e.s.d.'s (except the e.s.d. in the dihedral angle between two l.s. planes) are estimated using the full covariance matrix. The cell e.s.d.'s are taken into account individually in the estimation of e.s.d.'s in distances, angles and torsion angles; correlations between e.s.d.'s in cell parameters are only used when they are defined by crystal symmetry. An approximate (isotropic) treatment of cell e.s.d.'s is used for estimating e.s.d.'s involving l.s. planes.
Refinement. Refinement of *F*^2^ against ALL reflections. The weighted *R*-factor *wR* and goodness of fit *S* are based on *F*^2^, conventional *R*-factors *R* are based on *F*, with *F* set to zero for negative *F*^2^. The threshold expression of *F*^2^ > σ(*F*^2^) is used only for calculating *R*-factors(gt) *etc*. and is not relevant to the choice of reflections for refinement. *R*-factors based on *F*^2^ are statistically about twice as large as those based on *F*, and *R*- factors based on ALL data will be even larger.

### Fractional atomic coordinates and isotropic or equivalent isotropic displacement parameters (Å^2^)

**Table:**

	*x*	*y*	*z*	*U*_iso_*/*U*_eq_	
O1	0.82546 (12)	0.06885 (18)	0.65807 (11)	0.0380 (4)	
O2	0.79548 (13)	0.0605 (2)	0.45935 (11)	0.0434 (5)	
H2	0.7623	0.0135	0.4958	0.065*	
O3	1.16014 (12)	0.3820 (2)	0.71051 (11)	0.0402 (5)	
H3	1.1965	0.4229	0.6744	0.060*	
O4	1.12577 (13)	0.3814 (2)	0.51152 (12)	0.0454 (5)	
C1	0.95888 (17)	0.2202 (2)	0.47435 (15)	0.0275 (5)	
C2	0.88498 (16)	0.1437 (2)	0.51643 (15)	0.0274 (5)	
C3	0.89706 (16)	0.1438 (2)	0.62776 (15)	0.0259 (5)	
C4	0.99324 (15)	0.2298 (2)	0.69505 (14)	0.0232 (5)	
C5	1.06929 (16)	0.3034 (2)	0.65361 (15)	0.0267 (5)	
C6	1.05442 (17)	0.3056 (2)	0.54165 (16)	0.0284 (5)	
C7	0.9473 (2)	0.2204 (3)	0.36334 (16)	0.0399 (6)	
H7A	0.9064	0.1254	0.3332	0.060*	
H7B	1.0229	0.2215	0.3525	0.060*	
H7C	0.9050	0.3144	0.3331	0.060*	

### Atomic displacement parameters (Å^2^)

**Table:**

	*U*^11^	*U*^22^	*U*^33^	*U*^12^	*U*^13^	*U*^23^
O1	0.0323 (8)	0.0499 (10)	0.0336 (9)	−0.0172 (7)	0.0120 (7)	0.0003 (7)
O2	0.0398 (9)	0.0616 (12)	0.0274 (9)	−0.0259 (8)	0.0069 (7)	−0.0044 (8)
O3	0.0339 (9)	0.0544 (11)	0.0309 (9)	−0.0219 (7)	0.0066 (7)	−0.0014 (8)
O4	0.0416 (9)	0.0618 (11)	0.0351 (10)	−0.0259 (8)	0.0146 (7)	−0.0008 (8)
C1	0.0264 (10)	0.0320 (12)	0.0243 (11)	−0.0007 (9)	0.0074 (8)	−0.0006 (9)
C2	0.0236 (10)	0.0315 (12)	0.0259 (12)	−0.0045 (9)	0.0047 (8)	−0.0013 (9)
C3	0.0227 (10)	0.0269 (11)	0.0286 (12)	0.0003 (8)	0.0080 (8)	0.0034 (8)
C4	0.0223 (10)	0.0247 (11)	0.0227 (11)	0.0019 (8)	0.0064 (8)	0.0009 (8)
C5	0.0220 (10)	0.0289 (11)	0.0274 (12)	−0.0033 (8)	0.0040 (8)	−0.0019 (9)
C6	0.0260 (11)	0.0299 (12)	0.0313 (12)	−0.0024 (9)	0.0112 (9)	0.0018 (9)
C7	0.0424 (13)	0.0499 (15)	0.0291 (13)	−0.0063 (11)	0.0125 (10)	−0.0024 (11)

### Geometric parameters (Å, º)

**Table:**

O1—C3	1.223 (2)	C2—C3	1.499 (3)
O2—C2	1.335 (2)	C3—C4	1.452 (3)
O2—H2	0.8200	C4—C5	1.348 (3)
O3—C5	1.325 (2)	C4—C4^i^	1.476 (4)
O3—H3	0.8200	C5—C6	1.502 (3)
O4—C6	1.223 (2)	C7—H7A	0.9600
C1—C2	1.344 (3)	C7—H7B	0.9600
C1—C6	1.445 (3)	C7—H7C	0.9600
C1—C7	1.496 (3)		
			
C2—O2—H2	109.5	C3—C4—C4^i^	119.81 (18)
C5—O3—H3	109.5	O3—C5—C4	121.01 (18)
C2—C1—C6	117.14 (18)	O3—C5—C6	116.50 (17)
C2—C1—C7	123.62 (18)	C4—C5—C6	122.46 (17)
C6—C1—C7	119.24 (18)	O4—C6—C1	122.71 (19)
O2—C2—C1	120.65 (18)	O4—C6—C5	117.24 (18)
O2—C2—C3	115.94 (16)	C1—C6—C5	120.04 (17)
C1—C2—C3	123.41 (17)	C1—C7—H7A	109.5
O1—C3—C4	122.79 (19)	C1—C7—H7B	109.5
O1—C3—C2	117.97 (17)	H7A—C7—H7B	109.5
C4—C3—C2	119.23 (17)	C1—C7—H7C	109.5
C5—C4—C3	117.65 (18)	H7A—C7—H7C	109.5
C5—C4—C4^i^	122.51 (19)	H7B—C7—H7C	109.5

Symmetry code: (i) −*x*+2, *y*, −*z*+3/2.

### Hydrogen-bond geometry (Å, º)

**Table:**

*D*—H···*A*	*D*—H	H···*A*	*D*···*A*	*D*—H···*A*
O2—H2···O4^ii^	0.82	2.03	2.770 (2)	150
O3—H3···O1^iii^	0.82	2.03	2.7658 (19)	150

Symmetry codes: (ii) *x*−1/2, *y*−1/2, *z*; (iii) *x*+1/2, *y*+1/2, *z*.
